# Visual Perception of Moisture Is a Pathogen Detection Mechanism of the Behavioral Immune System

**DOI:** 10.3389/fpsyg.2020.00170

**Published:** 2020-02-13

**Authors:** Kazunori Iwasa, Takanori Komatsu, Ayaka Kitamura, Yuta Sakamoto

**Affiliations:** ^1^Department of Educational Psychology, Faculty of Education, Shujitsu University, Okayama, Japan; ^2^Department of Frontier Media Science, School of Interdisciplinary Mathematical Sciences, Meiji University, Tokyo, Japan; ^3^Emotions and Clinical Psychology Laboratory, Shujitsu University, Okayama, Japan

**Keywords:** behavioral immune system, disease avoidance, pathogen detection, visual moisture perception, psychophysics

## Abstract

The behavioral immune system (BIS) includes perceptual mechanisms for detecting cues of contamination. Former studies have indicated that moisture has a disgusting property. Therefore, moisture could be a target for detecting contamination cues by the BIS. We conducted two experiments to examine the psychophysical basis of moisture perception and clarify the relationship between the perception of moisture and the BIS. We assumed that the number of high luminance areas in a visual image provided optical information that would enable the visual perception of moisture. In two experiments, we presented eight images of dough that contained different amounts of moisture as experimental stimuli. The amount of moisture shown in the images was increased in eight steps, from 28.6 to 42.9% of the total weight of the dough. In Experiment 1, the images were randomly presented on a computer display, and the participants (*n* = 22) were asked to rank the images in the order of the visually perceived moisture content. In Experiment 2, the participants (*n* = 15) completed pairwise comparisons based on the perceived moistness of the images. Furthermore, to examine the BIS responses, the participants rated the strength of disgust evoked by the stimuli, their motivation to avoid touching the stimuli, and the estimated magnitude of the risk of contamination by physical contact with the stimuli. The results indicated that the moisture content and the numbers of high luminance areas in the images accurately predicted the perception of moisture, suggesting that the detection of visual moisture was highly accurate, and the optical information served as an essential perceptual cue for detecting moisture. On the other hand, the BIS responses peaked in response to stimuli having approximately 33 to 39% moisture content. These results show that objects containing a moderate amount of moisture could be the target of visually detecting pathogens by the BIS.

## Introduction

Infectious diseases are significant threats to our survival. The physiological immune system (PIS) protects us from these threats by resisting and eliminating pathogens in our body. One cost of this adaptive function is the sustained physiological burden on the body (Sheldon and Verhulst, 1996). The PIS is activated by the invasion of the body by pathogens. Therefore, humans are already at risk for infection by the time that the PIS starts to function. The behavioral immune system (BIS) is a motivational system that is oriented to the goal of avoiding diseases (Schaller and Park, 2011) and compensates for the limitations of the PIS by preventing physical contact with pathogens. The BIS is a psychological process consisting of several components, including a perceptual system for detecting pathogens, evaluating the threat of disease, and initiating avoidance behavior. An essential component of this motivational system is disgust, which has been considered to be an emotion for disease-avoidance (Matchett and Davey, 1991; Oaten et al., 2009; Tybur and Lieberman, 2016).

The concept of BIS is widely accepted today; however, the underlying psychological basis of the BIS has not been sufficiently clarified to date. Specifically, research focusing on the pathogen detecting process is relatively rare (Murray and Schaller, 2016). The perceptual detection of pathogens is positioned in the first phase of the psychological model of the BIS (Tybur and Lieberman, 2016). This suggests that the perceptual detection of pathogens plays a role in triggering the drive for later processing. There is evidence that perceptible cues suggesting the need to protect the self from disease can activate the BIS (Faulkner et al., 2004; Ackerman et al., 2009). It is also pointed out that emotional conditions and cognitive contexts might influence the detection process (Miller and Maner, 2012; Chan et al., 2016; Hunt et al., 2017), suggesting that this process works interactively with other components of the BIS. Therefore, it is necessary to clarify the psychological basis of the perceptual system for detecting pathogens to understand these interactions.

Specific studies have investigated the nature of the perceptual aspect of the BIS. Hunt et al. (2017) used psychophysical methods and reported that the induction of disgust enhanced tactile sensitivity such that it lowered the threshold for detecting tactile stimuli. Our skin is an anatomical barrier against pathogens. Therefore, skin lesions facilitate the intrusion of pathogens into the body and increase the risk of infections, suggesting that increased skin sensitivity might serve as a precursor to self-grooming behavior, which can decrease the risk of infection. Chan et al. (2016) found similar effects of disgust on the olfactory threshold. Odors can provide information about potential environmental hazards like food decay. Moreover, body products, which are regarded as disgust elicitors, are malodorous. In general, oral or tactile contact with such products that might contain pathogens is a risk to our health. Enhanced olfactory perceptual sensitivity facilitates pathogen detection and risk avoidance. The abovementioned studies have successfully investigated the perceptual aspect of BIS by using psychophysical methods. The purpose of psychophysics is to describe relationships between stimuli and psychological phenomena and identify mechanisms underlying these relationships, which was also the purpose of the current study. Therefore, we used psychophysical methods in this study.

The detection of pathogens by the BIS is not always accurate. Pathogens are microscopic, and they cannot be directly perceived by human beings. The detection process of the BIS consists of likelihood estimation using perceptible cues (Tybur and Lieberman, 2016). According to Schaller and Park (2011), this estimation is biased in favor of making false-positive judgments. This could be because although false-positive judgments in estimating pathogenicity can lead to subjective suffering and social dysfunctions due to excessive avoidance behavior and disgust, false-negative judgments might directly lead to a life crisis, and the false-positive bias is more advantageous for survival. Therefore, pathogenicity estimation by the BIS is tuned to a state of high sensitivity. As a result, the risk of infection can be overestimated when there are perceptual cues suggestive of pathogenicity, even in the absence of an actual risk of infection. This bias in the detection process is called *the smoke detector principle* (Nesse, 2005; Schaller and Park, 2011). The studies mentioned above (Chan et al., 2016; Hunt et al., 2017) also show that pathogen detection sensitivity fluctuates according to the state of activation of the BIS, which helps our survival, although it may cause psychosocial problems. For example, the fear of contamination is characterized by overestimating the pathogenicity of stimuli in the environment. Previous studies have indicated that the fear of contamination is one of the commonest themes in obsessive-compulsive disorder (McKay, 2006; Deacon and Olatunji, 2007), suggesting that the overestimating pathogenicity might be related to severe emotional and behavioral problems. Therefore, elucidating the process of detecting pathogens could potentially develop our understanding of psychopathologies, including obsessive-compulsive disorder.

### Current Study

This study investigated the psychophysical basis of visual moisture perception. Generally, warm water containing nutrients is known to promote the propagation of miscellaneous bacteria. Therefore, physical contact with this type of water might carry the risk of contracting infectious diseases. As a result, there could be survival value in the ability to visually detect moisture without using touch. Previous studies have demonstrated that moisture can cause reactions of disgust (Oum et al., 2011; Thibodeau, 2016). These findings indicate that the sense of wetness might be a phenomenological cue used by the BIS to estimate pathogenicity. However, we cannot directly calculate the moisture content of an object from visual information. Therefore, it is reasonable to assume that humans use perceptible optical information to estimate the moisture content of an object.

**FIGURE 1:**
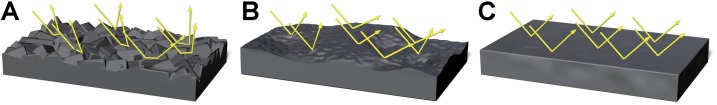
Images representing the difference in surface shape of the dough and light direction due to changes in moisture content. Arrays represent light direction. A rough surface reflects light in irregular directions **(A)**. A relatively smooth and wet surface reflects light at angles that are closer to the incident rays **(B)**. A smooth and liquid-state surface reflects light at angles that are nearly identical to incident rays **(C)**.

We focused on perceived moistness as a visible cue used for the detection of pathogens by the BIS. Organic materials containing abundant nutrients that absorb water, such as feces or food paste, easily acquire pathogenicity through bacterial growth. These materials tend to change their surface shape based on the amount of water they contain. For example, the surface of dough containing a small amount of water is rough and relatively matt. However, when the amount of water in the dough increases, the dough becomes smoother and gelatinous, which further increases the water content until it becomes flat and liquid (Figure 1).

The law of reflection states that the surface condition of an object defines the angle of reflection (Bennett and Porteus, 1961). Figure 1 shows the nature of reflection under different surface conditions. If the surface of the dough is rough, the incidental rays of light will strike areas that are inclined at different angles to each other, and therefore the reflected light rays would be diffused in irregular directions. However, if the surface is smooth and wet, the angle of the reflected light would be closer to the incidental rays. If the surface shape of liquid-like dough is flat, the angles of reflected light are nearly identical to the incidental rays. These characteristics change the perceived images of a light source when observed from a given angle to the object. Perceived images arising from a rougher surface might be segmented, whereas perceived images arising from a liquid-state surface might be more intact. Therefore, we predicted that the moisture content of an object could be estimated by the number of areas with high luminance. Based on this prediction, we focused on the number of high luminance areas contained in a stimulus image as a perceptible optical cue for the visual perception of moisture.

We conducted two experiments to clarify the process of visually detecting pathogenicity by the BIS. In Experiment 1, we used a simple ranking task consisting of sorting multiple images by quantifying the water content in the descending order of perceived moistness to evaluate the accuracy of visually estimating moisture. We also tested whether perceived moistness could be predicted by the number of high luminance areas in a stimulus image. Moreover, we investigated the BIS responses, including disgust, estimation of contamination risk, and the motive for avoidance behavior of stimuli, to clarify the amount of moisture that is used as a cue of pathogens. In Experiment 2, the replicability of the result of Experiment 1 was tested by using a pairwise comparison focusing on perceived moistness. Moreover, in both experiments, we used self-reports to assess the BIS responses directed toward the stimuli.

## Experiment 1

We investigated the psychophysical basis of visual moisture perception and its relationship with the BIS responses in Experiment 1.

### Methods

#### Participants

Female undergraduate students (*n* = 22, Mean age 20.46 years, *SD* = 1.97) participated in Experiment 1. The participants were recruited via an email invitation that explained the aims of the study and provided a brief description of the experimental procedure, the ethics of the study, and described the reward given to participants after completing the experiment (500 Japanese Yen, or approximately US$ 4.5).

#### Apparatus

Participants individually completed the experiment in a laboratory. Stimulus images were randomly placed in a 4 × 2 array and presented on a color-calibrated IPS-TFT LCD monitor (ColorEdge CG247, Eizo Nanao Corp., Tokyo, Japan). All responses were written and recorded on paper by the participants themselves. Participants sat in a chair that was placed approximately 60 cm from the display. However, no physical restrictions were placed on the distance between participants and the stimuli. Moreover, participants were able to observe the stimuli freely without being constrained by a time limit. The participants pressed the right key of the keyboard to move to the next ranking and rating task after completing each ranking and rating task. The entire experiment was controlled by the Microsoft PowerPoint program (Microsoft Corp., Redmond, WA, United States).

#### Materials

We presented pictures of dough (flour mixed with water) as the experiment stimuli such that the dough shown in each picture had a different moisture content. The moisture content was controlled using the following procedure. First, we mixed 90 g of flour with 10 g of black cocoa powder on a paper plate to color the dough for the easy identification of high luminance regions. Then, we added 2.5cc of water and mixed the dough using a plastic spatula. We made images of the stimuli by photographing the dough after adding 40cc or more water because the dough did not mix well with less than 40cc of water. The background of the photographs was kept constant, and the light source was set at a 45° angle of incident. A digital camera with a macro lens (20 mega-pixels; Canon, Tokyo, Japan) was set at the reflection angle of 135°, such that the area of specular reflection fitted in the center of the image and the distance from the light source to the dough was 60 cm. We placed the camera 20 cm away from the dough to capture the texture of the surface of the dough at a high resolution. The acquisition of all the pictures was completed in approximately 1.5 h.

**FIGURE 2:**
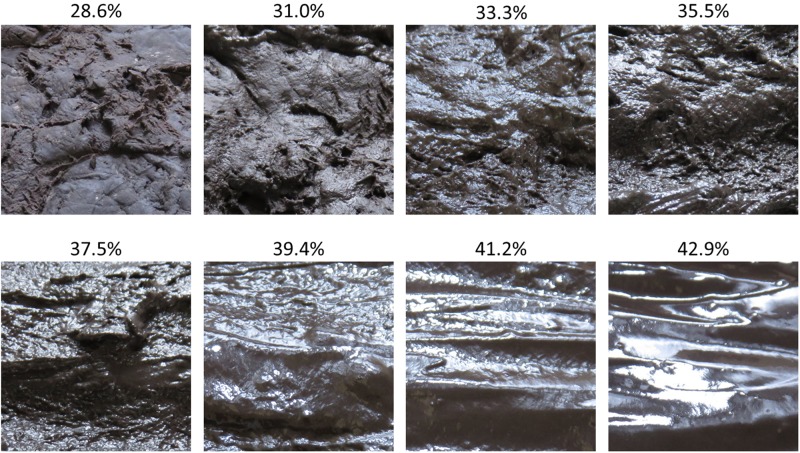
Images of dough used as experimental stimuli in this study. The percentages shown above each image indicate the ratio of water added to the total weight of dough. This ratio represents the estimated water content in each stimulus.

We photographed the dough with the identical degree of moisture on three occasions, each time changing the surface of the dough with a spatula. We also photographed the identical dough after blocking the light source, although these photographs were not used in the study. There was no significant change in the appearance of the dough when the water exceeded 75cc. Therefore, we selected the experimental stimuli from images having a water content of 40 to 75cc with the width of water content set at 5cc. One image that included the identical amount of water was randomly chosen as a stimulus. The moisture content of stimuli was quantified using the ratio of the amount of water added to the total weight of the dough. Therefore, the moisture content was not measured directly but estimated. Eight images having a moisture content ranging from 28.57 to 42.86% were selected as stimuli (Figure 2). We used these images after converting them to a grayscale. All images were trimmed to 512 × 512 pixels, centered on the specular reflection area of the light source.

#### Procedure

We examined the perceived moisture content of the stimuli by using a ranking task. The participants were asked to rank eight simultaneously displayed images in the order of increasing sense of moisture, to identify relative differences in appearance caused by changes in moisture content. The order of the stimuli on the display was randomized for each participant. The size of the presented images was 6 cm × 6 cm. Then, the participants rated the degree of disgust (“How disgusted do you feel about this object?”), motivation for avoiding physical contact (“How much do you want to avoid physical contact with this object?”), and perceived risk of contamination (“How much do you feel that you are likely to be contaminated by physical contact with this object?”) for each image. The stimuli were presented consecutively in the rating phase. The rating was conducted by using a 10-point Likert scale ranging from 1 (*extremely weak*) to 10 (*extremely strong*), with higher scores indicating stronger feelings, which were used to assess the BIS responses.

#### Data Analysis

**FIGURE 3:**
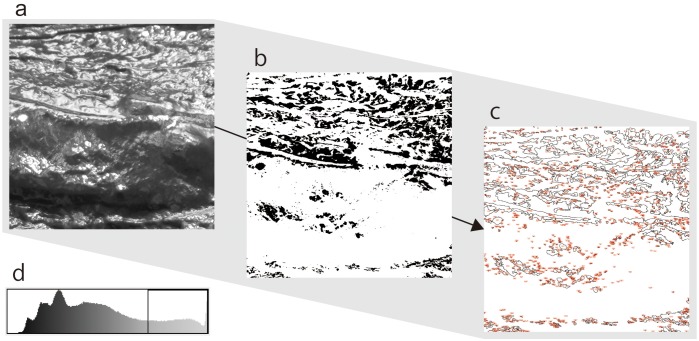
Sequence of particle analysis of this study. All pictures were gray scaled **(a)**, and then binarized according to thresholds of luminance values **(b)**. Each threshold of pictures was set at a value that is closest to the upper 20% in the luminance histogram **(d)**. Finally, the independent black particles in the binary image were counted **(c)**.

We conducted image analyses to count the number of high luminance areas included in the images by using the *particle analysis* function of an image analysis software (ImageJ 1.50: Schneider et al., 2012). Figure 3 depicts the sequence of analysis. First, we set the threshold of luminance values to extract areas of high luminance. The average values and the variance of luminance in each image were different. Therefore, a threshold value was set as the luminance value closest to the upper 20% of each image, instead of determining a threshold. Next, we converted all images into a binary form based on this threshold to extract high luminance areas. We converted all pixels in the images that had a luminance value under the threshold to white (luminance value = 255) and all pixels that had a luminance value above the threshold to black (luminance = 0). Finally, we counted the number of independent black particles (not pixels) contained in these images. Pearson product-moment correlation coefficient was calculated between the amount of water contained in the dough and the number of particles to identify the relationship between these variables. The result indicated a nearly linear correlation (*r* = −0.969), suggesting that the number of particles accurately reflected the amount of water in the stimuli.

We performed a series of regression analyses to clarify relationships between the variables examined in this study. First, we regressed the sense of moisture by the moisture content and the number of high luminance particles to clarify the psychophysical basis of visual moisture perception. Next, we regressed each BIS response by the amount of water to clarify the amount of moisture that is used as a cue of pathogens. We first applied linear regression analyses in these analyses. If the fitness of the linear model was insufficient, then we explored other methods such as polynomial regression analysis for curve fitting. Moreover, we adopted identical analyses in Experiment 1 and 2 to confirm the replicability of the results.

### Results and Discussion

Table 1 shows the descriptive statistics of all variables used in Experiment 1. We used the following scaling procedure for the sense of moisture. First, we inverted the rank values judged by the participants such that larger the value, the stronger was the sense of moisture. Next, we averaged these values for each stimulus and used these averaged values as scale values of the sense of moisture. Furthermore, we computed the standard errors for each stimulus. The procedure for calculating standard errors of scale values has not been established in Guilford’s (1954) normalized-rank method, which is a well-known scaling method of ranked values. In the present study, we applied the above procedure by giving priority to reporting interval estimates.

**TABLE 1:** The water content, number of high luminance particles, and descriptive statistics of self-reported variables of each image used in used in Experiment 1.

**Images**	**Water content**	**Number of particles**	**Sense of moisture**		**Disgust**		**Avoidance**		**Contamination**	
						
			**Mean**	***SD***	**Mean**	***SD***	**Mean**	***SD***	**Mean**	***SD***
No.1	0.286	3986	1.09	0.43	2.95	1.81	4.50	2.18	5.05	2.19
No.2	0.310	2593	2.05	0.49	4.50	2.04	5.77	2.33	6.73	2.21
No.3	0.333	2170	3.50	0.80	5.55	3.19	6.64	3.17	7.95	2.52
No.4	0.355	1759	4.00	0.76	5.91	2.86	6.77	2.91	7.73	2.68
No.5	0.375	1522	4.64	1.22	5.27	2.43	6.77	2.43	8.09	2.37
No.6	0.394	1194	6.05	0.38	5.18	3.29	6.82	3.05	8.27	2.29
No.7	0.412	851	6.68	0.78	4.50	3.04	6.14	2.87	7.55	2.76
No.8	0.429	436	8.00	0.00	4.09	3.13	5.59	3.20	7.14	2.83

**FIGURE 4:**
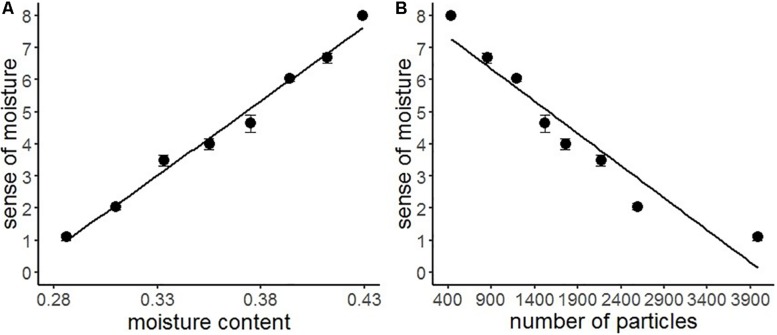
Regression analyses of sense of moisture by moisture content **(A)** and number of high luminance particles **(B)** with the regression line of Experiment 1. Bars represent standard errors.

We conducted a series of regression analyses to examine the psychophysical basis of sense of moisture (Figure 4). Results indicated that the water content accurately predicted the subjective sense of moisture (*R*^2^ = 0.99, *F*(1,6) = 227.30, *p* < 0.001), suggesting that the visual perception of moisture accurately reflected the relative amount of water contained in the stimuli. Furthermore, particles with high luminance in the images accurately predicted the sense of moisture (*R*^2^ = 0.91, *F*(1,6) = 69.49, *p* < 0.001), indicating that the distribution of high luminance particles on the object surface could be used as a perceptual cue for visually evaluating moisture.

**FIGURE 5:**
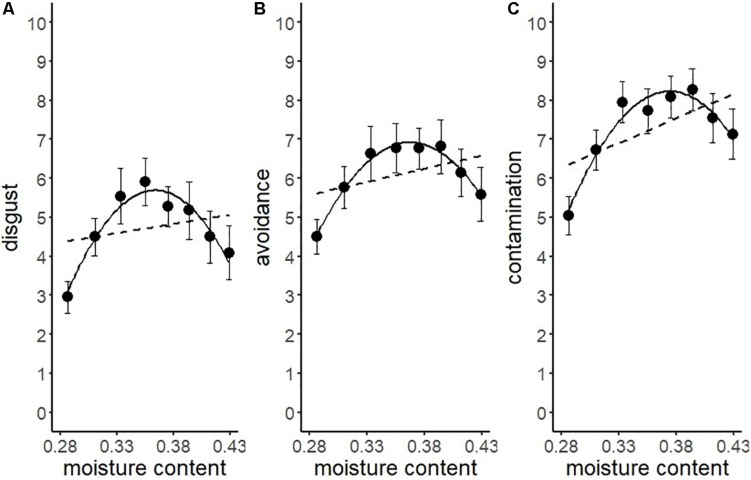
Linear versus quadratic regression of disgust **(A)**, motivation for avoiding physical contact **(B)**, and perceived risk of contamination **(C)** in Experiment 1. Broken lines represent the linear equation, and the solid line represents the quadratic equation. Bars represent standard errors.

We conducted another series of regression analysis to examine the BIS response to the stimuli (i.e., disgust, motivation for avoidance, and the estimation of the risk of contamination). Results showed that linear models did not fit well (*R*^2^ = 0.09, 0.03, 0.25, respectively). We inferred from the shape of the distribution that the relationship between each BIS response and water content approximated a quadratic curve (Figure 5). Therefore, we conducted quadratic multinomial regression analyses and compared the fitness of the quadratic models with linear models by using the Bayesian Information Criterion (*BIC*). The results indicated that the quadratic curve model provided a better fit for all the variables (Table 2). The quadratic curve showed an upward convex shape, indicating that moderate amounts of water (i.e., from 33.3 to 39.4%) tended to result in peak BIS responses. The omnibus effect size of water content was larger for contamination risk estimation and moderate for disgust and the motivation for avoidance (Table 2). These results suggested that optical information reflecting a moderate amount of water could be used as a perceptual cue for the detection of pathogens by the BIS.

**TABLE 2:** Summary of the regression analyses conducted in Experiment 1.

**Variables**	***BIC***		**Quadratic coefficient of quadratic equation**	**Effect size of water content**	
			
	**Linear**	**Quadratic**		**η^2^**	**95% CI**
Disgust	26.41	6.78	–431.546	0.091	0.001–0.146
Avoidance	23.07	–6.19	–358.717	0.062	0.000–0.105
Contamination	24.98	6.89	–395.479	0.139	0.029–0.205

**BIC* is Bayesian Information Criterion of linear and quadratic equations for each variable. The quadratic coefficient of quadratic equations and η^2^ are shown for summarizing the shapes of the quadratic curve.*

## Experiment 2

Experiment 1 indicated that the moisture content and the number of particles accurately predicted the sensation of moisture. Furthermore, the ratings of disgust, avoidance, and the perceived risk of contamination peaked for a moderate amount of moisture. We conducted a second experiment using a different methodology to test the replicability of the findings of Experiment 1.

## Methods

### Participants

Female undergraduate students (*n* = 15, Mean age 20.93 years, *SD* = 2.34) participated in Experiment 2. We recruited all participants via an emailed invitation that explained our research aims and provided a brief description of the experimental procedure, ethics, and reward for participation. No participants in Experiment 2 had taken part in Experiment 1. The participants received 500 Japanese Yen as a reward for taking part in the experiment.

### Material and Procedure

The stimuli used in Experimental 2 were the identical eight pictures of dough used in Experiment 1. In Experiment 2, we used the paired comparison method (Thurstone, 1927), which is one of the most common methods used in psychophysics experiments. The nature of a stimulus can be quantified by using this method and on a specific axis of evaluation. The purpose of this study was to quantify the perceived moistness of stimuli, and the paired comparison method was ideal for this purpose. We used the Bradley-Terry (BT) model (Bradley and Terry, 1952; Bradley, 1984; Turner and Firth, 2012) to convert the paired comparison data to a psychophysical rating scale, because the BT model can estimate confidence intervals more robustly than the Thurstone’s Case V model (Tsushima et al., 2016). We developed the paired comparison task in this study as follows: The eight images of the dough were combined to construct 28 stimulus pairs. These stimulus pairs were presented one at a time, such that the images were placed on the left and right sides. The size of the presented images was 16.14 cm × 16.14 cm. Each pair of stimuli was presented twice by interchanging left and right images to control for the influence of the presentation position. As a result, 56 paired comparisons were presented in one block in four blocks, resulting in 224 trials. Therefore, the participants performed the paired comparison judgment eight times for each stimulus pair. Since there were 15 participants in the experiment, the total number of comparison judgment for each pair was 120 judgments. The presentation order of the stimulus pairs was randomized for each block. The participants were requested to choose the image that gave them the strongest feeling of moistness. Eight practice trials were conducted before the experiment, in which the participants chose the more beautiful image from two images of landscapes.

The participants completed the task using nearly identical apparatus as in Experiment 1. We used a chinrest for all participants during Experiment 2 to fix the distance from the display and the participant’s face with the distance set at 60 cm. The task presentation was controlled by PsychoPy version 1.90.3 (Peirce et al., 2019). Responses were obtained by using a keyboard, such that the left image was selected by pressing the F key, and the right image was selected by pressing the J key. When the key was pressed, it automatically shifted to the next stimulus pair. As in Experiment 1, we asked the participants to rate the strength of disgust, motivation for avoiding physical contact, and the perceived risk of contamination for each image using a 10-point Likert scale.

The eight images for each item were presented in random order. This rating procedure was repeated four times to obtain a stable rating for these items. The mean ratings for each image were used in the analysis.

### Results and Discussion

**FIGURE 6:**
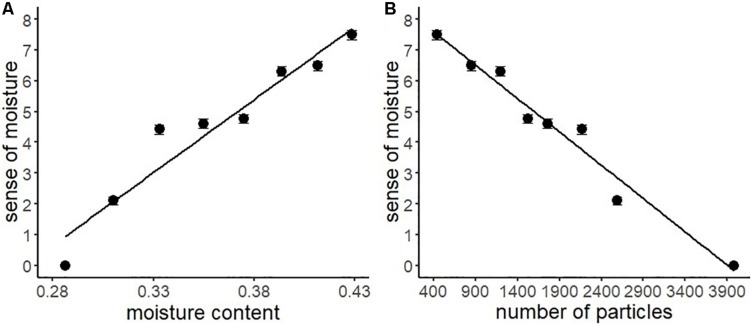
Regression analyses of the sense of moisture by moisture content **(A)** and the number of high luminance particles **(B)** with the regression line of Experiment 2. Bars represent standard errors.

Table 3 shows the descriptive statistics of all the variables used in Experiment 2. According to the above-mentioned data analysis plan, we conducted a series of regression analyses of psychophysical data of visual moisture perception (Figure 6). The result of regressing the sense of moisture by water content indicated that the water content accurately predicted the sense of moisture (*R*^2^ = 0.93, *F*(1,6) = 77.17, *p* < 0.001). Furthermore, we regressed the sense of moisture by the number of high luminance particles (*R*^2^ = 0.97, *F*(1,6) = 179.00, *p* < 0.001). The result indicated that the number of particles also accurately predicted the sense of moisture, which replicated the results of Experiment 1.

**TABLE 3:** The water content, number of high luminance particles and descriptive statistics of self-reported variables of each image used Experiment 2.

**Images**	**Water content**	**Number of particles**	**Sense of moisture**		**Disgust**		**Avoidance**		**Contamination**	
						
			**BT score**	***SE***	**mean**	***SD***	**mean**	***SD***	**mean**	***SD***
No.1	0.286	3986	0.00	0.00	5.17	3.26	5.80	3.35	4.93	3.27
No.2	0.310	2593	2.11	0.13	5.10	2.83	6.10	2.88	5.68	2.78
No.3	0.333	2170	4.42	0.15	5.65	2.41	6.45	2.78	6.12	2.30
No.4	0.355	1759	4.61	0.15	5.73	2.43	6.75	2.42	6.27	2.19
No.5	0.375	1522	4.77	0.15	5.58	2.25	6.63	2.58	6.35	2.16
No.6	0.394	1194	6.31	0.15	5.42	2.88	6.28	3.07	5.98	2.59
No.7	0.412	851	6.48	0.15	4.80	2.55	6.18	3.12	5.82	2.65
No.8	0.429	436	7.50	0.16	4.32	2.84	5.42	3.22	5.48	2.81

**FIGURE 7:**
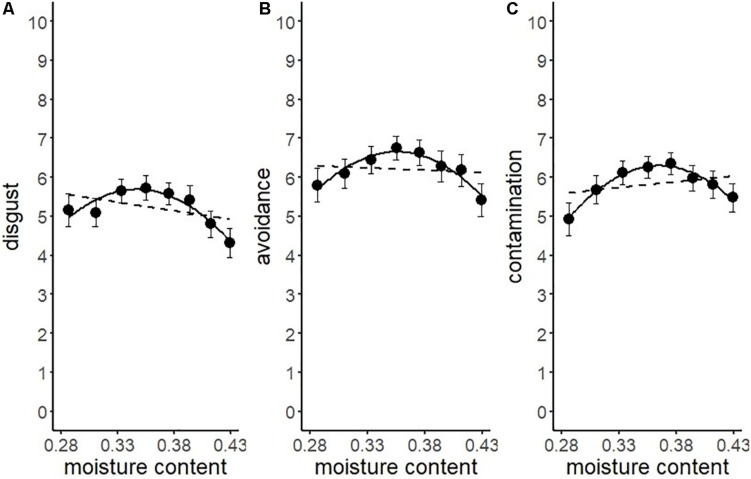
Linear versus quadratic regression of disgust **(A)**, motivation for avoiding physical contact **(B)**, and perceived risk of contamination **(C)** in Experiment 2. Broken lines represent the linear the equation, and solid lines represent the quadratic equation. Bars represent standard errors.

Another series of regression analysis indicated that the relationships between the BIS responses (disgust, motivation for avoidance, and the estimation of the risk of contamination) and water content, fitted a quadratic curve (Figure 7 and Table 4). The quadratic coefficients indicated that the curves were relatively gentle, and all omnibus effect sizes of water content were smaller than in Experiment 1 (Tables 2, 4). Similar to Experiment 1, these results supported the description of the BIS responses as a quadratic function of water content. However, it also suggested that factors other than the water content affected the magnitude of the BIS responses. The participants were exposed to the stimuli more frequently in Experiment 2 than in Experiment 1, which might have resulted in the habituation of emotional elements in the BIS response. From a different perspective, contrast adaptation might have influenced the results of Experiment 2. Contrast adaptation refers to the phenomenon in which prolonged viewing of a stimulus with a specific contrast reduces the perceptual and neural sensitivity to a subsequent stimulus with a similar contrast (Blakemore and Campbell, 1969; Smirnakis et al., 1997; Solomon et al., 2004). The stimuli used in this study were stained black, and therefore, the contrast with the reflective area was generally high. We can speculate that continuous exposure to high contrast stimuli caused contrast adaptation to the corresponding contrast band. Recent study revealed that temporal contrast adaptation can alter the visual discomfort of flicker stimuli (Yoshimoto et al., 2019). The relationship between spatial contrast adaptation and visual discomfort has not been clarified to date. Nevertheless, we cannot rule out that such a perceptual change might have affected the strength of the BIS responses. Another explanation of the difference between the results of the two experiments might be related to individual variations in the intensity of the BIS responses including disgust emotion (Haidt et al., 1994; Van Overveld et al., 2006; Duncan et al., 2009). We did not control for individual differences in these experiments, which might have been a confounding factor. In future research, it would be necessary to consider possible individual differences in BIS responses.

**TABLE 4:** Summary of the regression analyses conducted in Experiment 2.

**Variables**	***BIC***		**Quadratic coefficient of quadratic equation**	**Effect size of water content**	
			
	**Linear**	**Quadratic**		**η^2^**	**95% CI**
Disgust	14.36	1.77	–195.140	0.027	0.000–0.047
Avoidance	14.33	–1.57	–203.167	0.019	0.000–0.034
Contamination	15.01	–5.21	–212.980	0.027	0.000–0.047

**BIC* is Bayesian Information Criterion of linear and quadratic equations for each variable. The quadratic coefficient of quadratic equations and η^2^ are shown for summarizing the shapes of the quadratic curve.*

In sum, the results of Experiment 2 generally replicated the findings of Experiment 1. However, the effect sizes of water content on the BIS responses were smaller than in Experiment 1.

## General Discussion

In this study, we examined the psychophysical basis of visual moisture perception to elucidate the process of perceptually detecting pathogens by the BIS. Two experiments indicated that the visual perception of moisture could accurately estimate the relative amount of water contained in perceived objects. Also, it was shown that the number of high luminance particles distributed on the surface of a stimulus image could be used as a perceptual cue for visually evaluating the moisture content. The BIS responses consistently approximated the quadratic function of water content, although the omnibus effect sizes of water content on the BIS responses varied in the two experiments. Moreover, the BIS responses peaked for stimuli with intermediate amounts of moisture. These findings suggest that humans can evaluate environmental stimuli by using their surface textures and identify stimuli that might be contaminated because such stimuli would seem disgusting, and possibly pathogenic. This study identified the characteristics of the BIS when visually detecting the possible existence of pathogens in an object.

Nevertheless, the evaluation of pathogenicity demonstrated in this study does not correctly reflect actual risks. According to the literature (Troller and Christian, 1978; Barbosa-Cánovas et al., 2007), bacterial growth depends on the *water activity*, which is defined by the ratio of vapor pressures of the solution and solvent. This definition suggests that higher amounts of water contained in an object results in higher water activity. Bacteria and germs are known to breed well when water activity is higher than their indigenous threshold. In other words, there would be sufficient free water available to bacteria and germs for breeding in an environment with high water activity. Such microbial breeding is directly linked to spoilage and the risk of infections. This suggests that an increase in water content would not reduce the risk of contamination at least within the middle to the higher range of water content, as was the case in the present study. However, our results showed that the BIS responses fluctuated in an inverted U-shape with the increase in water content, suggesting that the participants in this study underestimated the risk of stimuli containing too much moisture. As mentioned above, the estimation of pathogenicity by the BIS tends to be biased in favor of false-positive results. Nevertheless, the results of this study were not consistent with this notion because underestimating the risk of contamination for very moist stimuli would have resulted in false negative-results.

There are some possible explanations for the discrepancy between the actual and the estimated risk observed in this study. Firstly, it is known that the cognitive context plays a crucial role in determining the emotional responses toward moist objects (Iwasa and Komatsu, 2015). For example, the moistness of vegetables or fruits is sometimes recognized as a sign of freshness, whereas moistness was evaluated as disgusting in the present study. This could be because the appearance of stimuli with an intermediate amount of moisture that was used in this study shared similarities with disgust elicitors such as feces, while stimuli with low and high moisture might be seen as other objects such as certain types of food. The perception of this similarity might have acted as the cognitive context for strengthening negative responses. Therefore, it is possible that moistness is interpreted in combination with properties of perceived objects, rather than by itself. It is suggested that the influence of such top-down processing should be examined in future research. Especially, asking what the stimulus looked like might also help to examine this possibility. Secondly, the results of this study can be explained by the variability of the spatial frequency of stimuli. Many studies have suggested that high amplitude images in a specific spatial frequency range cause aversive response (e.g., Conlon et al., 2001; Fernandez and Wilkins, 2008; O’Hare and Hibbard, 2011; Cole and Wilkins, 2013; Sasaki et al., 2017). This effect is robust and could be a potential factor in explaining the results of this study. However, investigating these possibilities was not within the scope of this study, and they remain to be investigated in future studies.

There are several limitations to this study. Firstly, we used only eight mixtures of dough as experimental stimuli, which limits the generalizability of our findings, because it is unclear whether the findings can be applied to materials other than dough. Sawayama et al. (2017) demonstrated that the surface color of wet objects looks more saturated, which suggests that perceptible cues of moisture might depend on the type of material. Furthermore, the number of stimuli used in these two experiments was small, which makes it difficult to conclude if the results reflect the general characteristics of dough or characteristics specific to the stimuli. This limitation can be solved by expanding the types of materials and the number of stimuli that are used in future experiments. Secondly, we applied only one image statistic, which is the number of high luminance areas based on the simple principle of reflection. Our results showed that this simple image statistic worked well for predicting the participant’s perception of visual moisture. However, it is possible that the prediction could have been made more robust by adding other image statistics, such as components of spatial frequency, the number of low and medium luminance areas, and the skew of the luminance histogram (Motoyoshi et al., 2007), among others, which is a possibility that should be addressed in the future. More simply, the pictures used in this research differed in their surface shape and streaks. These differences could not be controlled because we photographed the actual dough. Manipulating the luminance distribution of a 3D rendered image of the same object could be a solution for controlling these potential confounding effects. Thirdly, we suggested in the introduction that visual moisture perception might represent a process for detecting pathogens that have implications for psychopathology, which is potentially a productive possibility. However, this study did not provide any empirical findings on this topic. It is suggested that the relationship between individual differences in visual moisture perception and the BIS responses should be addressed in future studies. Moreover, examining the influences of psychopathology, such as obsessive-compulsive disorder, on visual moisture perception could be an exciting possibility, which could provide clinical benefits in for assessment and treatment of psychological disorders. Finally, we did not determine the sample sizes of each experiment in advance. The present research was based mainly on model fitting for psychophysical variables. The evaluation of the fitness of regression models can be more accurate when the sample sizes and the number of trials are optimally designed prior to the experiments. There are some suggestions about optimal design for paired comparison model (Graßhoff et al., 2004; Graßhoff and Schwabe, 2008). Designing experiments based on such suggestions is crucial for future studies.

## Conclusion

The results of this study showed that visual perception of moistness was accurate, and predicted by simple optical information. The BIS responses fluctuated in an inverted U-shape with the increasing water content in stimuli. These findings provided the first empirical support to the assumption that visual moisture perception serves as a part of the pathogen detection process of the BIS. On the other hand, this study raised new questions about underestimating the risk of contamination in stimuli showing a high content of water. The cognitive misestimation indicated by this finding, which is inconsistent with the smoke detection principle of the BIS, suggests the need for further research.

To the best of our knowledge, this is the first study to focus on the pathogen detection function related to visually perceiving moisture. Future research is needed to investigate detailed features of this function and its underlying mechanisms, including top-down processing. Progress in such research is expected to answer questions such as: What visual cues do people use for detecting the risk of infection? What psychological mechanisms enable the visual detection of cues suggestive of pathogens? What cognitive processes facilitate estimating the risk of infections and the biases in this process? Moreover, how do these cognitive processes affect our behavior and health? Exploring these questions will increase the sophistication of the BIS theory and its practical implications.

## Data Availability Statement

The datasets and stimuli used in this study are available in the Open Science Framework: https://osf.io/5quj9/.

## Ethics Statement

This study was reviewed and approved by the IRB of the Shujitsu University, in Japan. Written informed consent was obtained from all the participants before participation in the study. Participation was voluntary, and participants were informed that they could withdraw from the study at any time without providing a reason.

## Author Contributions

KI and TK conceived and designed the study and interpreted the results. KI, AK, and YS developed the experimental stimuli and collected the data. KI analyzed the data. KI, AK, YS, and TK reviewed and edited the manuscript, approved the final manuscript in the current form, and agreed to submit the manuscript for publication in the frontiers in Psychology.

## Conflict of Interest

The authors declare that the research was conducted in the absence of any commercial or financial relationships that could be construed as a potential conflict of interest.
